# A Pilot Analysis of Bioparameters in Patients with Dyspepsia Accompanied by Abdominal Hardness: An Exploration of *Damjeok* Syndrome Rooted in Traditional Medicine

**DOI:** 10.3390/healthcare13182307

**Published:** 2025-09-15

**Authors:** Yun-Seo Lim, Chang-Gue Son, Jin-Hee Lee, Sung-don Yang, Daeui Park, Gi-Hwan Rho, Gyu-Ho Choi, Seo-Hyung Choi

**Affiliations:** 1 Department of Integrative Medicine, Wooje Research Institute for Integrative Medicine, Seoul 06200, Republic of Korea; dlawkgud@weedahm.com (Y.-S.L.); goodyang@weedahm.com (S.-d.Y.); 2 Department of Internal Medicine, Weedahm Korean Medicine Hospital, Seoul 06185, Republic of Korea; rghdoc71@naver.com (G.-H.R.); hiro9582@hanmail.net (G.-H.C.); 3 Department of Internal Medicine, Daejeon Oriental Hospital of Daejeon University, Daejeon 35235, Republic of Korea; ckson@dju.ac.kr; 4 Department of Integrative Medicine, Chungju Weedahm Integrative Hospital, Chungju 27498, Republic of Korea; jinheelee80@gmail.com; 5 Department of Predictive Toxicology, Korea Institute of Toxicology, Daejeon 34114, Republic of Korea; daeui.park@kitox.re.kr

**Keywords:** *Damjeok* syndrome, abdominal hardness, functional gastrointestinal disorders, gut–brain axis, parasympathetic activity, 5-hydroxyindoleacetic acid, heart rate variability

## Abstract

**Background**: A subset of patients with chronic dyspepsia exhibits palpable upper abdominal hardness and systemic symptoms like headache, chest discomfort, neck/shoulder stiffness, fatigue, and depression. In traditional Korean medicine (TKM), this symptom complex is referred to as *Damjeok* syndrome (痰积症候群, DJS). Although DJS is frequently observed in TKM practice, it lacks a clear case definition and biological mechanism, limiting its integration in gastroenterology research and evidence-based practice. Clarifying its clinical and biological features is essential to understand its pathophysiology and clinical significance. **Methods**: This case–control study aimed to characterize DJS by comparing 16 female patients diagnosed with DJS and 15 age-matched healthy females as controls. A female-only cohort was selected to reflect the higher prevalence of chronic dyspepsia in women and reduce biological variability. Clinical characteristics and potential DJS-specific biomarkers were evaluated through complete blood count (CBC), serum biochemical tests, heart rate variability (HRV) for autonomic function, and plasma 5-hydroxyindoleacetic acid (5-HIAA), a serotonin metabolite linked to gastrointestinal motility and autonomic regulation. **Results**: The DJS group had a mean disease duration of 58.0 ± 46.2 months, with epigastric fullness and underlying abdominal hardness as primary complaints. Postprandial distress syndrome (PDS) was the most common (43.8%) dyspepsia subtype, often combined with epigastric pain syndrome (EPS). Extra-gastrointestinal symptoms such as headache/fatigue (87.5%) and anxiety/depression (81.3%) were highly prevalent. Neutrophil counts were significantly lower in the DJS group (*p* = 0.01), while other hematological or biochemical markers showed no differences (*p* > 0.1). HRV analysis revealed decreased parasympathetic activity (RMSSD and HF, *p* < 0.1), and plasma 5-HIAA levels were significantly elevated compared to healthy controls (*p* = 0.01). **Conclusions**: DJS aligns with functional gastrointestinal disorders (FGIDs), sharing psychosomatic symptoms and reduced parasympathetic activity, suggesting gut–brain axis dysregulation. However, distinct features like palpable upper abdominal hardness and elevated plasma 5-HIAA levels indicate that DJS may represent a unique subtype within the category of FGIDs. These findings highlight the need for larger, well-designed studies to further elucidate the pathophysiology of DJS.

## 1. Introduction

Functional gastrointestinal disorders (FGIDs) affect a large portion of the global population, accounting for approximately 40% of individuals, and present with chronic or recurrent gastrointestinal symptoms without detectable structural abnormalities [1]. FGIDs, including representative conditions such as functional dyspepsia (FD) and irritable bowel syndrome (IBS), are classified as disorders of gut–brain interaction, involving altered motility, visceral hypersensitivity, immune dysfunction, microbial imbalance, and abnormal processing of the central nervous system [2,3]. Among these, FD is the most prevalent upper gastrointestinal FGID, with a global pooled prevalence of 8.4% [4], and it presents with persistent symptoms such as pain or burning in the epigastric region, early satiety, and postprandial fullness [5]. Various beneficial treatments for FD include anti-acid therapy, prokinetics, and central neuromodulators [6]. However, the relatively high recurrence rate following symptomatic relief from these regimens presents a clinical issue. For instance, approximately 20% of patients with FD reportedly experience relapse within 3 months after a 4-week treatment with a proton pump inhibitor (PPI) [7], and 25% relapse at one year after treatment with acotiamide [8]. Moreover, about 25% of FD patients do not respond to two or more standard treatments, indicating refractory FD [9]. These clinical challenges in treating FD reflect its heterogeneous and multifactorial pathophysiology, which remains incompletely understood [10]. Consequently, about 80% of patients with FD have a chronic disease course with significantly impaired quality of life (QoL) [11,12,13], comparable to that of patients with diseases such as gastroesophageal cancer [14], myocardial infarction [15], and chronic kidney disease [16]. Specifically, patients with refractory FD experience more than twice the prevalence of anxiety, depression, and sleep disorders, and incur higher medical expenses compared to those with non-refractory FD [9].

On the other hand, traditional Korean medicine has long recognized patients with severe and chronic dyspepsia, characterized by a hardened pattern palpated in the upper abdomen, and has diagnosed them with the pattern of *Damjeok* (痰积) [17,18]. *Damjeok* (痰积) refers to the accumulation and solidification (积) of phlegm (痰), a viscous waste product derived from an impaired digestive system [18,19]. While phlegm primarily accumulates in the gastrointestinal tract, it is also believed to circulate throughout the body, contributing to a diverse range of symptoms [20,21]. These include gastrointestinal symptoms such as epigastric discomfort, nausea, and upper abdominal pain, as well as systemic manifestations like fatigue, chest tightness, and dizziness [22]. *Damjeok* is characterized by the adhesive and solidifying properties of phlegm [23], resulting in a hardened mass in the upper abdomen that can be palpated and used as a key diagnostic sign. From a modern biomedical perspective, the broad and diverse symptomatology of *Damjeok* may be understood as a syndrome rather than a single disease entity. Based on this, we identified a subset of patients with severe and chronic dyspepsia who presented with a distinctive pattern of abdominal hardness on palpation and classified this group as a novel clinical entity termed *Damjeok* syndrome (DJS) [18].

Along with unique abdominal characteristics, patients with DJS exhibit gastrointestinal symptoms centered around the epigastric region and a range of extra-gastrointestinal symptoms such as headache, dizziness, chest discomfort, neck and shoulder stiffness, fatigue, anxiety, and/or depression [17,24]. While DJS shares some overlapping features with FD, particularly in epigastric symptoms and psychological manifestations [25], the key distinguishing clinical characteristic of DJS is abdominal hardness, which serves as the primary target for both diagnosis and treatment [26]. Treatment of DJS typically involves traditional Korean medical therapies combining herbal medicine aimed at resolving phlegm accumulation and external physical therapies such as acupuncture, moxibustion, manual therapy, and vibratory stimulation targeting abdominal rigidity [27,28,29]. Notably, therapeutics based on DJS have demonstrated effectiveness in improving chronic and refractory dyspepsia [27].

Despite its growing use in clinical settings, DJS remains poorly defined within biomedical frameworks, with limited biological data supporting its distinctiveness. Systematically investigating DJS by combining traditional diagnostic features with objective laboratory markers will allow for mechanistic insights into the syndrome, validate the traditional diagnostic concept, and strengthen its recognition in contemporary clinical practice. Therefore, this study aimed to investigate the key clinical and biological characteristics of patients diagnosed with DJS, based on diagnostic criteria developed from a review of traditional medical literature. This preliminary investigation into DJS may offer a novel clinical perspective for managing cases of refractory dyspepsia insufficiently addressed by current diagnostic and therapeutic paradigms, while also laying the groundwork for understanding its pathophysiology.

## 2. Materials and Methods

### 2.1. Study Design and Participants

This case–control pilot study was conducted at the Weedahm Korean Medicine Hospital in Seoul, South Korea, from 17 November 2022 to 20 January 2023. The study protocol was approved by the Institutional Review Board of Weedahm Korean Medicine Hospital (approval no. WD-KH-2022-001), and written informed consent was obtained from all participants prior to enrollment. A total of 31 female participants (16 patients with DJS and 15 healthy controls) were included.

Participants were recruited through convenience sampling, with DJS patients recruited from the hospital outpatient clinic and healthy controls recruited from the local community. The sample size was determined based on feasibility and reference to commonly recommended ranges for pilot studies [30,31], in line with the exploratory nature of this case–control pilot study. Female participants aged 40 to 60 years were selected to reflect the higher prevalence of chronic dyspepsia in women, to minimize biological variability, and to represent the typical demographic observed in DJS clinical practice (Figure S1) [32].

Patients with DJS were selected from hospital outpatients based on predefined diagnostic criteria, and all had chronic symptoms persisting for more than six months or recurring after temporary improvement. Healthy controls were recruited from the local community, and 15 were enrolled after eligibility was confirmed through screening to ensure the absence of digestive symptoms and upper abdominal hardness. The case and control groups were matched by age to minimize confounding by demographic factors.

The inclusion criteria for the DJS group required participants to meet all three diagnostic criteria for DJS, as summarized in Table 1 [18]. For the HC group, participants were required to have neither significant digestive symptoms nor detectable upper abdominal hardness upon palpation. The exclusion criteria for all participants were as follows [33,34]: (1) a history of definite organic gastrointestinal diseases such as peptic ulcer, severe reflux esophagitis, gastric cancer, or pancreaticobiliary disease, confirmed by upper endoscopy performed within the past 6 months, (2) a history of significant systemic disorders requiring ongoing medication, including diabetes, hypertension, or dyslipidemia, (3) a history of alcohol or drug abuse, and (4) pregnancy or breastfeeding.

All participants underwent standardized diagnostic assessments during the screening process, which included structured history taking and abdominal palpation performed by trained physicians. In this process, baseline parameters of the study population, including sex, age, and body mass index (BMI), were recorded along with the symptoms and abdominal findings. Following this, blood samples were collected and heart rate variability (HRV) tests were conducted once for each participant.

### 2.2. DJS Criteria and Diagnosis

The diagnostic criteria for DJS consist of three core symptoms and signs, as summarized in Table 1. Briefly, (1) the patient should report dyspepsia in the form of epigastric fullness, pain, or burning, which significantly affects daily and social activities at level 3 or 4. These symptoms are not fully explained by endoscopic findings and persist for over 6 months or recur even after temporary improvement; (2) the patient should exhibit abdominal hardness palpated by physicians in the upper abdomen at level 3 or 4; and (3) the patient should present with one or more of six extra-gastrointestinal symptoms, such as headache/dizziness, chest discomfort, neck and shoulder stiffness, fatigue, and anxiety/depression. These symptoms should have newly appeared or worsened after the onset of dyspepsia.

### 2.3. Assessment of Epigastric Symptoms

Dyspepsia-related epigastric symptoms, such as epigastric fullness, pain, or burning, were assessed using a self-report questionnaire, and their severities were evaluated using a 5-point Likert scale (0 to 4). Briefly, 0 (none) indicates no symptom; 1 (mild) indicates occasional symptoms with slight discomfort; 2 (moderate) indicates frequent or prolonged symptoms requiring relief; 3 (severe) indicates symptoms causing substantial discomfort and hindrance in social activities; and 4 (very severe) indicates continuous symptoms severely interfering with daily activities, causing extreme discomfort and distress. The total epigastric symptom score was calculated as the sum of the severity scores for epigastric fullness, pain, and burning, and this total score was used for further analyses.

### 2.4. Classification into FD Subgroups

Regarding the FD-related epigastric symptoms, participants were classified into three subgroups according to ROME IV FD criteria. The postprandial distress syndrome (PDS) is characterized by postprandial fullness or early satiation, while epigastric pain syndrome (EPS) is characterized by pain or burning in the epigastric region, and a mixed group (PDS + EPS) is also defined [34].

### 2.5. Abdominal Examination

For the assessment of abdominal hardness, participants lay supine with their upper abdomen exposed, knees slightly flexed, and arms resting at their sides to minimize abdominal wall tension. The physician, standing beside the bed, initiated palpation using a two-handed technique around the midpoint between the xiphisternal junction and the umbilicus in a circular pattern [35].

**Figure 1:**
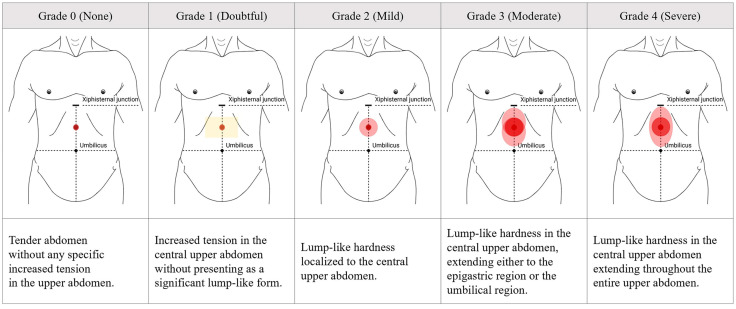
Grading of upper abdominal hardness presented in *Damjeok* syndrome. Images for 5-scale grades ranged from 0 to 4 according to the severity of upper abdominal hardness assessed by palpation. This figure was originally created by the authors based on the diagnostic elements of *Damjeok* syndrome, particularly the physical features of upper abdominal hardness, as described in a previous literature review [18].

Abdominal hardness was assessed on a 5-grade scale (Figure 1): 0 (none) for a tender upper abdomen; 1 (doubtful) for increased tension in the central upper abdomen; 2 (mild) for lump-like hardness localized to the central upper abdomen; 3 (moderate) for central lump-like hardness extending either superiorly into the epigastric region or inferiorly toward the umbilical region; and 4 (severe) for central lump-like hardness extending throughout the entire upper abdomen. Cases with excessive rectus abdominis tension or severe distension were excluded.

All abdominal examinations were performed independently by two trained Korean medicine doctors, each possessing a minimum of three years of clinical experience, who had completed standardized internal training based on the grading protocol for upper abdominal hardness (Figure 1) to ensure diagnostic reproducibility [36,37]. The final grading was determined only when both physicians reached consensus.

### 2.6. Assessment of Extra-Gastrointestinal Symptoms

Six extra-gastrointestinal symptoms, including headache/dizziness, chest discomfort, neck and shoulder stiffness, fatigue, and anxiety/depression, were considered core extra-gastrointestinal symptoms of DJS if they emerged or worsened following the onset of dyspepsia, leading to noticeable discomfort rather than being transient.

### 2.7. Analysis of Complete Blood Counts (CBC) and Biochemistry

Peripheral blood samples were collected by venous puncture after fasting for at least 8 h. The complete blood count (CBC) was analyzed using an XN-9000 hematology analyzer (Sysmex Corporation, Kobe, Japan), while the erythrocyte sedimentation rate (ESR) was measured using TEST 1 (Alifax, Padua, Italy).

Serum levels of total protein, albumin, aspartate aminotransferase (AST), alanine aminotransferase (ALT), alkaline phosphatase (ALP), gamma-glutamyl transferase (γ-GT), total bilirubin, direct bilirubin, creatinine, and blood urea nitrogen (BUN), as well as high-sensitivity C-reactive protein (hs-CRP) and four lipid profile items, were measured using the Cobas 8000 analyzer (Roche Diagnostics, Mannheim, Germany). Hemoglobin A1c (HbA1c) levels were measured using the Cobas c 513 (Roche Diagnostics, Mannheim, Germany). The estimated glomerular filtration rate (eGFR) was determined based on the Chronic Kidney Disease Epidemiology Collaboration (CKD-EPI) formula [38].

Plasma levels of 5-hydroxyindoleacetic acid (5-HIAA), the primary metabolite of serotonin (5-HT), were measured using liquid chromatography with tandem mass spectrometry (Agilent, Santa Clara, CA, USA). Given the high biological variability of direct 5-HT measurements in blood, 5-HIAA was used as a more stable and reliable surrogate marker of serotonin metabolism [39]. In addition to fasting for at least 8 h, participants were instructed to avoid serotonin-rich foods such as bananas, avocados, walnuts, eggplant, and tomatoes, as well as serotonergic medications, including acetaminophen and tryptophan supplements for at least 48 h before blood sampling, according to established protocols to reduce pre-analytical variability [40,41,42].

### 2.8. Heart Rate Variability Test

HRV was measured for 5 min using the SA-3000 device (Medicore, Seoul, Republic of Korea), with three electrodes placed on the wrists and the left ankle of the participants [43]. All measurements were performed in a quiet, temperature-controlled room between 10:00 a.m. and 12:00 p.m. to ensure standardization [44]. Participants were instructed to avoid strenuous activity, caffeine, and smoking before the test [45,46,47]. Before the measurement, participants lay in a supine position on a bed, having removed any metal objects from their bodies and were allowed to rest for 10 min to stabilize autonomic activity [48].

Values for the standard deviation of NN intervals (SDNN) and the root mean square of successive differences (RMSSD) in the time domain were used, along with the powers of high frequency (HF, 0.15–0.4 Hz) and low frequency (LF, 0.04–0.15 Hz) in the frequency domain, with the low-to-high frequency power ratio (LHR, LF power/HF power) calculated from the device.

### 2.9. Statistical Analysis

Categorical variables, such as the presence of clinical features, were presented as frequencies and percentages. Continuous variables, including the grade/score of clinical features, blood-derived parameters, and HRV indices, were expressed as means ± standard deviations, and significant differences between the DJS and HC groups were assessed using an independent two-sample *t*-test. Correlations between the epigastric symptom scores of the DJS group and 5-HIAA levels, as well as HRV indices, were analyzed using Pearson’s correlation coefficient. Statistical analyses were performed using SPSS version 24.0 (SPSS Inc., Chicago, IL, USA), with significance set at *p* < 0.1 due to the exploratory nature and small sample size of this pilot study [49]. Formal corrections for multiple comparisons were not applied to preserve statistical power [50]. Data visualization was performed using GraphPad Prism version 5.01 (GraphPad Software Inc., San Diego, CA, USA).

## 3. Results

### 3.1. Clinical Characteristics of the Participants

Both healthy and DJS groups had a similar age (50.7 ± 2.4 vs. 49.9 ± 6.7 years), while BMI was significantly lower in the DJS group (21.0 ± 2.2 kg/m^2^) than in the control group (23.3 ± 3.4 kg/m^2^). Patients with DJS had an average illness duration of 58.0 ± 46.2 months, and a grade of abdominal hardness of 3.9 ± 0.3. They primarily complained of epigastric fullness (87.5%) followed by epigastric pain (43.8%) and epigastric burning (37.5%), which were categorized as PDS (43.8%), EPS (12.5%), and mixed-type (43.8%) FD, respectively.

Regarding the extra-gastrointestinal symptoms, most patients with DJS reported headache or dizziness (87.5%) and/or fatigue (87.5%), as well as neck and shoulder stiffness (81.3%), anxiety or depression (81.3%), and/or chest discomfort (75.0%), respectively (Table 2).

### 3.2. Complete Blood Count and Blood Biochemistry Profile

In the CBC profiles, the average RBC, WBC, and platelet counts, as well as hemoglobin and ESR levels, were within normal ranges in both healthy and DJS groups. Meanwhile, 43% of DJS patients had a low WBC count, and the absolute neutrophil count was significantly lower in the DJS group compared to the control group (2.3 ± 0.5 vs. 3.1 ± 0.8 10^3^/µL, *p* < 0.05).

Regarding blood biochemistry, liver and renal function parameters, including eGFR were within normal ranges, although eGFR was significantly lower in the DJS group (106.0 ± 6.6 vs. 97.6 ± 13.3 mL/min/1.73 m^2^, *p* < 0.05). In addition, no significant differences were observed in hs-CRP, HbA1c, and lipid parameters between the two groups (Table 3).

### 3.3. Heart Rate Variability Profile

**Figure 2:**
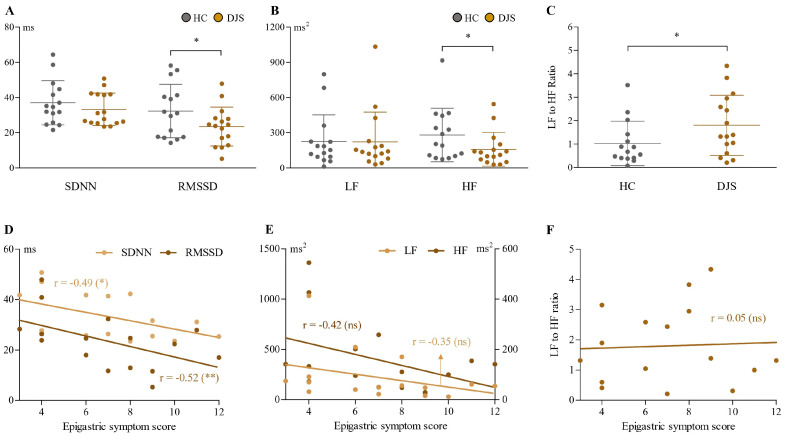
Analysis of HRV parameters. Time domain parameters (SDNN and RMSSD in ms) (**A**), frequency domain parameters (LF and HF in ms^2^) (**B**), and LF-to-HF ratio (**C**) were compared between the DJS and HC groups. In the DJS group, correlations between epigastric symptom scores and time domain (**D**), frequency domain (**E**), and LF-to-HF ratio (**F**) parameters were analyzed. Independent two-sample *t*-tests were used for group comparisons, and Pearson’s correlation coefficient was used to assess relationships within the DJS group. Statistical significance was set at *p* < 0.1; * indicates *p* < 0.1, ** indicates *p* < 0.05, and ns denotes not significant. SDNN, standard deviation of NN interval; RMSSD, root mean square of the successive differences; LF, low frequency; HF, high frequency; HC, healthy control; DJS, *Damjeok* syndrome.

Compared to healthy controls, the DJS group showed altered patterns in HRV parameters across both time and frequency domains (Figure 2A–C). In the time domain, SDNN values were slightly lower in the DJS group (33.29 ± 9.26 vs. 37.05 ± 12.53 ms), but the difference was not statistically significant. In contrast, RMSSD values were notably reduced in the DJS group (23.5 ± 11.03 vs. 32.32 ± 15.16 ms), meeting the predefined threshold for statistical significance (*p* < 0.1).

In the frequency domain, HF power was significantly lower in the DJS group (158.70 ± 143.39 vs. 281.50 ± 227.61 ms^2^), whereas LF power did not differ significantly between groups (222.80 ± 253.78 vs. 225.94 ± 226.46 ms^2^). The LF-to-HF power ratio was elevated in the DJS group compared to controls (1.80 ± 1.28 vs. 1.03 ± 0.95).

Further analyses within the DJS group revealed that the time domain parameters (both SDNN and RMSSD) showed a significant negative correlation with the epigastric symptom score, with a stronger correlation in RMSSD (r = −0.52, *p* < 0.05) (Figure 2D). The frequency domain (both LF and HF power) also exhibited a tendency toward a negative correlation with the epigastric symptom score (as a more prominent correlation in HF, r = −0.42, *p* = 0.1); however, these indices, including the ratio, did not reach statistical significance (Figure 2E,F).

### 3.4. Plasma 5-HIAA

**Figure 3:**
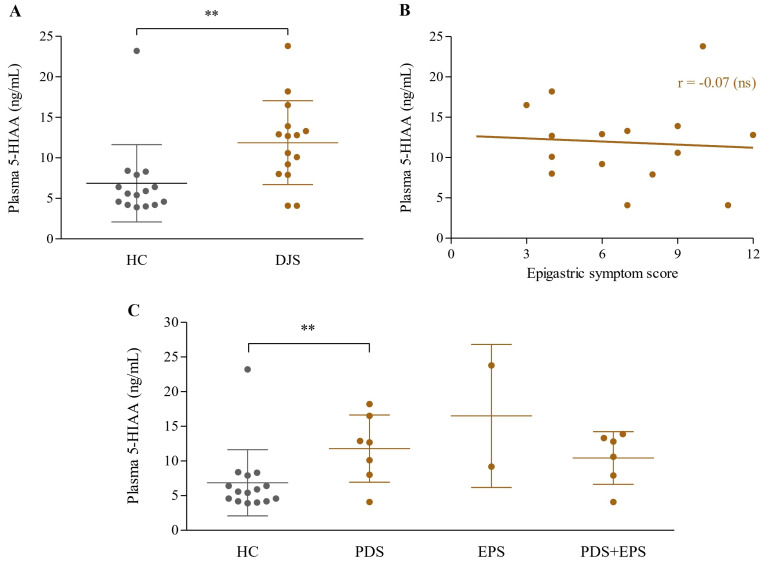
Analysis of plasma 5-HIAA levels. Plasma 5-HIAA concentrations (ng/mL) were compared between the HC and DJS groups (**A**), analyzed for correlation with epigastric symptom scores within the DJS group (**B**), and compared across FD subgroups (**C**). Comparisons between the two groups were conducted using independent two-sample *t*-tests, and correlations between continuous variables within the DJS group were analyzed using Pearson’s correlation coefficient. Statistical significance was set at *p* < 0.1; ** indicates *p* < 0.05, and ns denotes not significant. 5-HIAA, 5-hydroxyindoleacetic acid; HC, healthy control; DJS, *Damjeok* syndrome; PDS, postprandial distress syndrome; EPS, epigastric pain syndrome; FD, functional dyspepsia.

Patients with DJS exhibited significantly higher levels of plasma 5-HIAA compared to the control group (11.9 ± 5.2 vs. 6.9 ± 4.8 ng/mL, *p* = 0.01), while only the PDS group showed a significant difference (11.8 ± 4.9 ng/mL, *p* < 0.05) under subgroup analysis (Figure 3A,C).

When comparing plasma levels of 5-HIAA with the epigastric symptom score within the DJS group, no significant correlation was observed (r = −0.07, *p* = 0.8) (Figure 3B).

## 4. Discussion

Despite severe dyspepsia and high epigastric symptom scores persisting for about five years (Table 2), and in the absence of notable endoscopic findings, patients with DJS did not exhibit major abnormalities in hematological or metabolic parameters, including liver and renal function (Table 3). These findings align with the nature of FGIDs, in which patients often report severe gastrointestinal symptoms in the absence of detectable organic abnormalities [5]. The prominent symptom localization in the epigastric region, along with the chronic disease course, places DJS within the conceptual framework of FGIDs, particularly resembling upper gastrointestinal disorders such as FD in some clinical aspects [33]. However, DJS is distinguished from FD by the presence of a characteristic palpable hardness in the upper abdomen (Figure 1), a physical sign not addressed in the clinical evaluation of FD. In addition, patients with DJS frequently report a unique sense of epigastric fullness, often described as tightness or pressure, which is not featured in the FD diagnostic criteria [51]. Furthermore, while FD is known to accompany psychosomatic issues such as anxiety, depression, and fatigue [52,53,54], DJS involves a broader range of systemic manifestations, such as headache, chest discomfort, and musculoskeletal pain, which serve as prominent and defining features of the syndrome (Table 2).

Given the combination of chronic dyspeptic symptoms and diverse systemic manifestations, DJS may involve dysregulation of the gut–brain axis, a core framework in the pathophysiology of FGIDs [3]. Autonomic dysfunction has been well established as a key pathophysiological feature in many FGIDs, including FD and IBS [55]. We assessed autonomic function via HRV analysis to explore the biological basis of potential gut–brain dysregulation in DJS. HRV quantifies the temporal fluctuations between successive heartbeats, reflecting the complex regulatory balance between sympathetic and parasympathetic components of the autonomic nervous system [56]. It is typically assessed through time-domain parameters, such as SDNN and RMSSD, which quantify the amount of heart rate variability [57]. Besides time-domain analysis, frequency-domain parameters, including LF and HF power, represent distinct components of autonomic regulation by measuring the energy of signals within specific frequency ranges [58]. In our analysis, patients with DJS exhibited significantly reduced RMSSD and HF values compared to healthy controls (Figure 2A–C), indicating decreased parasympathetic activity [59].

Furthermore, both RMSSD and HF showed negative correlations with the severity of epigastric symptoms, suggesting that lower vagal tone may contribute to symptom exacerbation (Figure 2D,E). In contrast, LF values, which are relatively indicative of sympathetic modulation [60], were similar to those of the healthy controls, and were not associated with epigastric symptoms within the DJS group (Figure 2B,E). These findings are consistent with previous studies reporting reduced vagal activity in FD and gastroparesis, where impaired gastric emptying is a prominent feature [55]. Similarly, patients with DJS predominantly fell into the PDS type (Table 2), showing symptoms of postprandial fullness and early satiety usually related to delayed gastric emptying. Taken together, these results suggest that reduced parasympathetic tone may contribute to both the dyspeptic symptoms and the systemic features of DJS, potentially mediated through delayed gastric emptying. Furthermore, the characteristic upper abdominal hardness observed in DJS may physically hinder gastric motility, thereby exacerbating meal-related symptoms.

In this context, we focused on serotonin (5-HT) metabolism, a key neuromodulatory pathway involved in gastrointestinal motility, visceral hypersensitivity, and central nervous system function [61]. Given its central role in mutual communication between the gut and brain, alterations in serotonergic signaling have been implicated in the pathophysiology of FGIDs. They are thought to contribute to their frequent comorbidity with mood- and pain-related symptoms, including anxiety, depression, fatigue, headache, and non-cardiac chest discomfort [62,63,64]. However, direct measurement of 5-HT in the blood is often limited by high biological variability [39]. Therefore, we assessed plasma levels of 5-HIAA, a stable and quantifiable metabolite of serotonin, to better understand serotonergic activity in DJS. Interestingly, plasma 5-HIAA levels were significantly increased in the DJS group compared to the healthy controls (Figure 3A). This is in contrast to results in FD and IBS, where 5-HIAA levels have been reported to be consistently reduced regardless of subtype [65,66]. This distinctive biochemical pattern in DJS may be related to its unique clinical features, particularly the characteristic upper abdominal hardness. Since 5-HT release can be stimulated by intraluminal pressure and mechanical stretch within the gastrointestinal tract [66], it is conceivable that the focal abdominal rigidity induces increased serotonergic activity and subsequent metabolite accumulation. Recent experimental studies using in vitro and ex vivo models, including isolated enterochromaffin (EC) cells and gut organoids, have demonstrated that mechanical stimuli such as compression, shear force, and matrix stiffness can activate mechanosensitive EC cells via calcium influx, often through Piezo2 channels, leading to serotonin release [67,68]. These findings support the hypothesis that the characteristic abdominal hardness in DJS may act as a mechanical trigger that enhances serotonin turnover, resulting in elevated plasma 5-HIAA levels observed in this study. Although a direct causal relationship between abdominal hardness and serotonergic activity could not be established in this study, our strict inclusion of patients with consistently palpable epigastric hardness strengthens the plausibility of this mechanism. This raises the possibility that abdominal stiffness may not merely represent a clinical marker but may also contribute actively to the observed dysregulation in the gut–brain axis.

In addition to these neuroanatomic and serotonergic changes, we also observed significantly lower neutrophil counts in the DJS group compared to healthy controls (Table 3). This hematological finding was in accordance with the lower gene expressions associated with neutrophil extracellular trap (NET) formation observed in RNA sequencing analysis using peripheral blood from the DJS group (Park et al., in preparation) [69]. These data may indicate a reduced host defense capacity against infections or inflammation, making patients with DJS more susceptible to systemic health issues [70].

Taken together, the observed alterations in parasympathetic activity and serotonergic metabolism suggest that DJS involves dysregulation of the gut–brain axis, consistent with the core mechanism underlying FGIDs. However, the clinical and biochemical features of DJS, particularly the palpable upper abdominal hardness and elevated plasma 5-HIAA levels, differ from those typically observed in FD or IBS. These findings imply that while DJS shares fundamental pathophysiological elements with FGIDs, it may represent a distinct clinical entity within this spectrum. Although this pilot study could not confirm a direct mechanistic link between upper abdominal hardness and the identified biomarkers, the consistent presence of this localized rigidity suggests it may reflect or contribute to the autonomic and serotonergic dysregulation observed in DJS. Further investigation is needed to clarify whether this localized stiffness is merely a clinical marker or actively involved in the gut–brain axis dysfunction.

This study has several limitations. First, although it was designed as a pilot investigation, the small sample size and inclusion of only female participants limit the generalizability of the findings. Second, all biological parameters were assessed only at baseline, before any treatment, without follow-up evaluation after clinical improvement. Longitudinal data are needed to determine whether the observed abnormalities normalize with symptom resolution. Third, while the study focused on identifying biological features associated with DJS, it did not include a comparison group of patients exhibiting similar dyspeptic and systemic symptoms without abdominal hardness, limiting the ability to determine the specificity of the findings to DJS. Lastly, due to the exploratory nature of the study, we adopted a relaxed statistical threshold of *p* < 0.1 to detect potential trends. Although this approach is often employed in exploratory studies, the findings must be viewed cautiously given the elevated risk of type I error.

Despite these limitations, this is the first study to comprehensively examine the clinical characteristics, autonomic function, and blood-based biomarkers in patients with DJS. The findings offer preliminary biological support for DJS as a distinct clinical entity. They may contribute to the development of novel diagnostic and therapeutic strategies for patients with unexplained or refractory gastrointestinal symptoms. Future well-controlled studies with larger and more diverse cohorts, appropriate clinical comparison groups (e.g., FD patients with and without upper abdominal stiffness), and longitudinal follow-up are warranted to validate the present findings and further elucidate the pathophysiology of DJS.

## 5. Conclusions

This pilot study provides preliminary evidence that DJS, a clinical entity in traditional Korean medicine characterized by palpable upper abdominal hardness, may represent a distinct subtype within the category of FGIDs. Patients with DJS exhibited chronic epigastric symptoms without notable structural or routine biochemical abnormalities, aligning with the functional nature of FGIDs and overlapping partially with FD. However, unlike FD, DJS was defined by characteristic abdominal hardness and accompanied by prominent systemic involvement, as well as a unique sense of epigastric fullness. While reduced parasympathetic activity observed in DJS is consistent with patterns reported in FGIDs, the significantly elevated plasma 5-HIAA levels may reflect a distinct serotonergic feature. Based on these clinical and biological findings, we cautiously suggest that DJS could represent a potential subtype of gut–brain axis disorder, possibly associated with upper abdominal hardness. By interpreting traditional diagnostic features within a modern biomedical framework and integrating them with objective laboratory markers, this study provides mechanistic insight into DJS and supports its clinical relevance. Further studies with larger and more diverse cohorts including both sexes are warranted to confirm these findings and further elucidate the pathophysiology of DJS.

## Figures and Tables

**Table 1:** Case definition of *Damjeok* syndrome.

*Damjeok* syndrome must fulfill all the following 3 conditions:
Unexplained dyspepsia involving one or more of epigastric fullness, pain, or burning, significantly affecting social and daily activities. - A symptom that affects social and daily activities is equivalent to a score of 3 or 4 on a 0–4-point Likert scale *. - The sum of scores for epigastric fullness, pain, and burning should be 3 or higher. Abdominal hardness palpated in the upper abdomen centered around the midpoint of the xiphisternal junction and umbilicus, corresponding to Grade 3 or 4 (Figure 1). One or more of the following extra-gastrointestinal symptoms triggered or exacerbated by indigestion. Headache/Dizziness Chest discomfort Neck and shoulder stiffness Fatigue Anxiety/Depression

* 5 point (0–4) Likert scale: 0 (none) indicates no or transient symptom; 1 (mild) indicates occasional symptom with slight discomfort; 2 (moderate) indicates frequent or prolonged symptom requiring relief; 3 (severe) indicates symptom causing substantial discomfort and hindrance in social activities; and 4 (very severe) indicates continuous symptom severely interfering with daily activities, causing extreme discomfort and distress.

**Table 2:** Baseline demographic and clinical characteristics.

Characteristics	*Damjeok* Syndrome (n = 16)	Healthy Control (n = 15)	*p* Value
Female, n (%)	16 (100)	15 (100)	-
Age, year	49.9 ± 6.7	50.7 ± 2.4	0.69
BMI, kg/m^2^	21.0 ± 2.2	23.3 ± 3.4	0.04
Duration of illness, month	58.0 ± 46.2	0	-
Abdominal hardness			
Presence, n (%)	16 (100)	0 (0)	-
Grade (0–4)	3.9 ± 0.3	0	-
DJS symptom			
Epigastric symptom			
Epigastric fullness, n (%)	14 (87.5)	0 (0)	-
Epigastric pain, n (%)	7 (43.8)	0 (0)	-
Epigastric burning, n (%)	6 (37.5)	0 (0)	-
Total score (0–12)	7.0 ± 2.8	0	-
FD-based subtype			
PDS, n (%)	7 (43.8)	0 (0)	-
EPS, n (%)	2 (12.5)	0 (0)	-
PDS + EPS, n (%)	7 (43.8)	0 (0)	-
Extra-gastrointestinal symptom			
Headache/Dizziness, n (%)	14 (87.5)	0 (0)	-
Chest discomfort, n (%)	12 (75.0)	0 (0)	-
Neck and shoulder stiffness, n (%)	13 (81.3)	0 (0)	-
Fatigue, n (%)	14 (87.5)	0 (0)	-
Anxiety/Depression, n (%)	13 (81.3)	0 (0)	-

Mean ± standard deviation and *p* value were calculated using an independent two-sample *t*-test for continuous variables, and n (%) was used for categorical variables. BMI, body mass index; FD, functional dyspepsia; PDS, postprandial distress syndrome; EPS, epigastric pain syndrome. The study included only female participants to reduce biological variability and to focus on a population with a higher prevalence of chronic dyspepsia.

**Table 3:** Routine complete blood count and blood biochemistry analyses.

Parameter	Reference Range	DJS Group (n = 16)	HC Group (n = 15)	*p* Value
		Mean ± SD	Mean ± SD	
Complete blood count				
RBC (10^6^/µL)	3.7–4.7	4.4 ± 0.4	4.3 ± 0.3	0.57
Hb (g/dL)	11–15	13.4 ± 1.0	13.2 ± 1.0	0.67
WBC (10^3^/µL)	4–10	4.4 ± 1.0	5.2 ± 1.3	0.10
Neutrophil (10^3^/µL)	≥1.5	2.3 ± 0.5	3.1 ± 0.8	0.01
Lymphocyte (10^3^/µL)	≤4	1.7 ± 0.5	1.6 ± 0.6	0.65
Platelet (10^3^/µL)	150–370	262.6 ± 46.4	247.8 ± 66.7	0.52
ESR (mm/h)	≤25	12.8 ± 10.9	12.2 ± 7.3	0.86
Blood biochemistry				
hs-CRP (mg/L)	≤0.9	0.4 ± 0.3	0.5 ± 0.6	0.48
HbA1c (%)	≤5.6	5.5 ± 0.3	5.7 ± 0.5	0.15
Total-Cholesterol (mg/dL)	<200	208.2 ± 48.2	212.9 ± 37.4	0.76
HDL-Cholesterol (mg/dL)	>40	70.3 ± 17.1	72.7 ± 16.7	0.69
LDL-Cholesterol (mg/dL)	<100	125.3 ± 45.8	129.6 ± 35.2	0.77
Triglyceride (mg/dL)	<150	89.4 ± 32.9	76.7 ± 40.3	0.34
Total protein (g/dL)	6.6–8.7	7.5 ± 0.5	7.3 ± 0.2	0.23
Albumin (g/dL)	3.5–5.2	4.7 ± 0.3	4.6 ± 0.2	0.40
AST (U/L)	0–32	20.3 ± 4.4	18.9 ± 3.0	0.32
ALT (U/L)	0–33	13.4 ± 5.4	17.3 ± 8.1	0.12
ALP (U/L)	0–32	20.3 ± 4.4	18.9 ± 3.0	0.32
γ-GT (U/L)	6–42	17.4 ± 6.6	16.1 ± 5.6	0.56
Total bilirubin (mg/dL)	0.0–1.2	0.6 ± 0.2	0.5 ± 0.2	0.52
Direct bilirubin (mg/dL)	0.0–0.3	0.2 ± 0.1	0.2 ± 0.1	0.60
BUN (mg/dL)	6–20	14.0 ± 4.9	12.7 ± 3.1	0.38
Creatinine (mg/dL)	0.5–0.9	0.7 ± 0.1	0.6 ± 0.1	0.01
eGFR (mL/min/1.73 m^2^)	≥90	97.6 ± 13.3	106.0 ± 6.6	0.04

Mean ± standard deviation and *p* value were calculated using an independent two-sample *t*-test for continuous variables. ALT, aminotransferase; ALP, alkaline phosphatase; AST, aspartate aminotransferase; BUN, blood urea nitrogen; CRP, C-reactive protein; DJS, *Damjeok* syndrome; eGFR, estimated glomerular filtration rate; ESR, erythrocyte sedimentation rate; RBC, red blood cell; γ-GT, gamma-glutamyl transferase; Hb, hemoglobin; HC, healthy control; HDL, high-density lipoprotein; LDL, low-density lipoprotein; WBC, white blood cell.

## Data Availability

The data presented in this study are available on request from the corresponding author. The data are not publicly available due to ethical restrictions, as they involve human participants and contain potentially identifiable or sensitive health information.

## Supplementary Materials

The following supporting information can be downloaded at: https://www.mdpi.com/article/10.3390/healthcare13182307/s1, Figure S1. Age and sex distribution of patients with *Damjeok* syndrome. Age and sex distribution of 150 patients diagnosed with *Damjeok* syndrome. Female patients accounted for 71.3% (n = 107), with the largest proportion in their 60 s, followed by those in their 50 s. Data were obtained from retrospective chart review at Weedahm Korean Medicine Hospital. This study was approved by the Institutional Review Board of Weedahm Korean Medicine Hospital (IRB No. WD00003-21-CR-001).

## Abbreviations

The following abbreviations are used in this manuscript:

DJS	Damjeok syndrome
TKM	Traditional Korean medicine
FGID	Functional gastrointestinal disorder
RFD	Refractory functional dyspepsia
CBC	Complete blood count
5-HIAA	5-Hydroxyindoleacetic acid
HRV	Heart rate variability
RMSSD	Root mean square of successive differences
HF	High frequency
FD	Functional dyspepsia
PPI	Proton pump inhibitor
QoL	Quality of life
PDS	Postprandial distress syndrome
EPS	Epigastric pain syndrome
ESR	Erythrocyte sedimentation rate
AST	Aspartate aminotransferase
ALT	Alanine aminotransferase
ALP	Alkaline phosphatase
γ-GT	gamma-Glutamyl transferase
BUN	Blood urea nitrogen
hs-CRP	High-sensitivity C-reactive protein
HbA1c	Hemoglobin A1c
eGFR	Estimated glomerular filtration rate
CKD-EPI	Chronic Kidney Disease Epidemiology Collaboration
SDNN	Standard deviation of NN interval
LF	Low frequency
LHR	Low-to-high frequency
BMI	Body mass index
NET	Neutrophil extracellular trap

## References

[1] Black C.J., Drossman D.A., Talley N.J., Ruddy J., Ford A.C. (2020). Functional gastrointestinal disorders: Advances in understanding and management. Lancet.

[2] Drossman D.A. (2016). Functional Gastrointestinal Disorders: History, Pathophysiology, Clinical Features and Rome IV. Gastroenterology.

[3] Drossman D.A., Hasler W.L. (2016). Rome IV-Functional GI Disorders: Disorders of Gut-Brain Interaction. Gastroenterology.

[4] Lee K., Kwon C.I., Yeniova A., Koyanagi A., Jacob L., Smith L., Lee S.W., Rahmati M., Shin J.Y., Shin J.I. (2024). Global prevalence of functional dyspepsia according to Rome criteria, 1990–2020: A systematic review and meta-analysis. Sci. Rep..

[5] Schmulson M.J., Drossman D.A. (2017). What Is New in Rome IV. J. Neurogastroenterol. Motil..

[6] Talley N.J., Ford A.C. (2015). Functional Dyspepsia. N. Engl. J. Med..

[7] Meineche-Schmidt V., Talley N.J., Pap A., Kordecki H., Schmid V., Ohlsson L., Wahlqvist P., Wiklund I., Bolling-Sternevald E. (1999). Impact of functional dyspepsia on quality of life and health care consumption after cessation of antisecretory treatment. A multicentre 3-month follow-up study. Scand. J. Gastroenterol..

[8] Shinozaki S., Osawa H., Sakamoto H., Hayashi Y., Miura Y., Lefor A.K., Yamamoto H. (2017). Adherence to an acotiamide therapeutic regimen improves long-term outcomes in patients with functional dyspepsia. J. Gastrointest. Liver Dis..

[9] Jiang S.M., Jia L., Lei X.G., Xu M., Wang S.B., Liu J., Song M., Li W.D. (2015). Incidence and psychological-behavioral characteristics of refractory functional dyspepsia: A large, multi-center, prospective investigation from China. World J. Gastroenterol..

[10] Ford A.C., Moayyedi P., Black C.J., Yuan Y., Veettil S.K., Mahadeva S., Kengkla K., Chaiyakunapruk N., Lee Y.Y. (2021). Systematic review and network meta-analysis: Efficacy of drugs for functional dyspepsia. Aliment. Pharmacol. Ther..

[11] Madisch A., Andresen V., Enck P., Labenz J., Frieling T., Schemann M. (2018). The Diagnosis and Treatment of Functional Dyspepsia. Dtsch. Arztebl. Int..

[12] Shetty A.J., Balaraju G., Shetty S., Pai C.G. (2017). Quality of life in dyspepsia and its subgroups using EQ-5D (EuroQol) questionnaire. Saudi J. Gastroenterol..

[13] Hantoro I.F., Syam A.F., Mudjaddid E., Setiati S., Abdullah M. (2018). Factors associated with health-related quality of life in patients with functional dyspepsia. Health Qual. Life Outcomes.

[14] Xiao H., Bertwistle D., Khela K., Middleton-Dalby C., Hall J. (2023). Global retrospective analysis of clinician- and patient-reported clinical characteristics and humanistic burden of patients with gastroesophageal cancers on first-line treatment. BMC Cancer.

[15] Huber A., Oldridge N., Höfer S. (2016). International SF-36 reference values in patients with ischemic heart disease. Qual. Life Res..

[16] Finkelstein F.O., van Nooten F., Wiklund I., Trundell D., Cella D. (2018). Measurement properties of the Short Form-36 (SF-36) and the Functional Assessment of Cancer Therapy—Anemia (FACT-An) in patients with anemia associated with chronic kidney disease. Health Qual. Life Outcomes.

[17] Choi H.S., Kim J.K., Choi S.H. (2009). Recent Advances in Diagnosis of Gastrointestinal Disease. J. Soc. Korean Med. Diagn..

[18] Lim Y.S., Rho G.H., Choi G.H., Lee S.H., Choi S.H. (2023). A Literature Study on the Diagnostic Factors and Value as a Syndrome of Damjeok. J. Korean Med..

[19] Zhang Z., Hu J. (2016). Recent Advances and Perspective of Studies on Phlegm Syndrome in Chinese Medicine. Evid. Based Complement. Altern. Med..

[20] Greenwood M.T. (2017). Dysbiosis, Spleen Qi, Phlegm, and Complex Difficulties. Med. Acupunct..

[21] Park J., Choi T.J., Kang K.S., Choi S.H. (2021). The Interrelationships between Intestinal Permeability and Phlegm Syndrome and Therapeutic Potential of Some Medicinal Herbs. Biomolecules.

[22] Park J.S., Yang D.H., Kim M.Y., Lee S.C., Park Y.J. (2006). Development of Questionnire for Damum Patternization. J. Soc. Korean Med. Diagn..

[23] Zhu Z. (1962). Experience in diagnosis and treatment of Zhengjia in traditional Chinese medicine. Fujian J. Tradit. Chin. Med..

[24] Na B.J., Choi S.H. (2012). Clinical Analysis of the 991 Outpatients with Gastrointestinal Symptoms and Extra-gastrointestinal Symptoms. J. Korean Med..

[25] Wu J.C. (2012). Psychological Co-morbidity in Functional Gastrointestinal Disorders: Epidemiology, Mechanisms and Management. J. Neurogastroenterol. Motil..

[26] Lee Q.J., Lee Y.H., Shin T.M. (2006). The Development and Response Characteristic Analysis of Damjeok Diagnosis System Using Ultrasonic Sensor. Trans. Korean Inst. Electr. Eng. D.

[27] Rho G.H., Choi G.H., Lee S.H., Choi S.H., Noh H.M. (2022). The Effect of Korean Medical Complex Treatment on Functional Dyspepsia Patients: Through Measurement of Functional Dyspepsia Symptoms through NDI-K, Pressure Pain Threshold through an Algometer. J. Physiol. Pathol. Korean Med..

[28] Choi S.H. (2024). Damjeok Syndrome: Origin of All Disease.

[29] Cha E.S., Na Y.H. (2024). Treatment of Damjeok Syndrome in a Cooperative Way with Korean Medical Treatment and Visceral Manipulation. J. PMS.

[30] Kunselman A.R. (2024). A brief overview of pilot studies and their sample size justification. Fertil. Steril..

[31] In J. (2017). Introduction of a pilot study. Korean J. Anesthesiol..

[32] Napthali K., Koloski N., Walker M.M., Talley N.J. (2016). Women and functional dyspepsia. Womens Health.

[33] Tack J., Talley N.J., Camilleri M., Holtmann G., Hu P., Malagelada J.R., Stanghellini V. (2006). Functional gastroduodenal disorders. Gastroenterology.

[34] Stanghellini V., Chan F.K., Hasler W.L., Malagelada J.R., Suzuki H., Tack J., Talley N.J. (2016). Gastroduodenal Disorders. Gastroenterology.

[35] Bilal M., Voin V., Topale N., Iwanaga J., Loukas M., Tubbs R.S. (2017). The Clinical anatomy of the physical examination of the abdomen: A comprehensive review. Clin. Anat..

[36] Valentín-Mazarracin I., Nogaledo-Martín M., López-de-Uralde-Villanueva I., Fernández-de-Las-Peñas C., Stokes M., Arias-Buría J.L., Díaz-Arribas M.J., Plaza-Manzano G. (2021). Reproducibility and Concurrent Validity of Manual Palpation with Rehabilitative Ultrasound Imaging for Assessing Deep Abdominal Muscle Activity: Analysis with Preferential Ratios. Diagnostics.

[37] Ha N.Y., Ko S.J., Park J.W., Kim J. (2024). Development of a Standard Tool of Pattern Identification for Functional Dyspepsia: A Cross-Sectional Study from Korea. Healthcare.

[38] Levey A.S., Stevens L.A., Schmid C.H., Zhang Y.L., Castro A.F., Feldman H.I., Kusek J.W., Eggers P., Van Lente F., Greene T. (2009). A new equation to estimate glomerular filtration rate. Ann. Intern. Med..

[39] Tyce G.M. (1990). Origin and metabolism of serotonin. J. Cardiovasc. Pharmacol..

[40] Ewang-Emukowhate M., Subramaniam K., Lam F., Hayes A., Mandair D., Toumpanakis C., Grossman A., Nair D., Caplin M. (2023). Plasma or serum 5-hydroxyindoleacetic acid can be used interchangeably in patients with neuroendocrine tumours. Scand. J. Clin. Lab. Investig..

[41] Tohmola N., Johansson A., Sane T., Renkonen R., Hämäläinen E., Itkonen O. (2015). Transient elevation of serum 5-HIAA by dietary serotonin and distribution of 5-HIAA in serum protein fractions. Ann. Clin. Biochem..

[42] Lenchner J.R., Santos C. (2022). Biochemistry, 5 Hydroxyindoleacetic Acid. StatPearls.

[43] Kim G.M., Woo J.M. (2011). Determinants for heart rate variability in a normal Korean population. J. Korean Med. Sci..

[44] Catai A.M., Pastre C.M., Godoy M.F., Silva E.D., Takahashi A.C.M., Vanderlei L.C.M. (2020). Heart rate variability: Are you using it properly? Standardisation checklist of procedures. Braz. J. Phys. Ther..

[45] Barutcu I., Esen A.M., Kaya D., Turkmen M., Karakaya O., Melek M., Esen O.B., Basaran Y. (2005). Cigarette smoking and heart rate variability: Dynamic influence of parasympathetic and sympathetic maneuvers. Ann. Noninvasive Electrocardiol..

[46] Sondermeijer H.P., van Marle A.G., Kamen P., Krum H. (2002). Acute effects of caffeine on heart rate variability. Am. J. Cardiol..

[47] Perini R., Veicsteinas A. (2003). Heart rate variability and autonomic activity at rest and during exercise in various physiological conditions. Eur. J. Appl. Physiol..

[48] Ryan A.D., Larsen P.D., Galletly D.C. (2003). Comparison of heart rate variability in supine, and left and right lateral positions. Anaesthesia.

[49] Serdar C.C., Cihan M., Yücel D., Serdar M.A. (2021). Sample size, power and effect size revisited: Simplified and practical approaches in pre-clinical, clinical and laboratory studies. Biochem. Med..

[50] Rothman K.J. (1990). No adjustments are needed for multiple comparisons. Epidemiology.

[51] Futagami S., Yamawaki H., Agawa S., Higuchi K., Ikeda G., Noda H., Kirita K., Akimoto T., Wakabayashi M., Sakasegawa N. (2018). New classification Rome IV functional dyspepsia and subtypes. Transl. Gastroenterol. Hepatol..

[52] Goggins E., Mitani S., Tanaka S. (2022). Clinical perspectives on vagus nerve stimulation: Present and future. Clin. Sci..

[53] VanElzakker M.B. (2013). Chronic fatigue syndrome from vagus nerve infection: A psychoneuroimmunological hypothesis. Med. Hypotheses.

[54] Li H., Page A.J. (2022). Altered Vagal Signaling and Its Pathophysiological Roles in Functional Dyspepsia. Front. Neurosci..

[55] Ali M.K., Chen J.D.Z. (2023). Roles of Heart Rate Variability in Assessing Autonomic Nervous System in Functional Gastrointestinal Disorders: A Systematic Review. Diagnostics.

[56] McCraty R., Shaffer F. (2015). Heart Rate Variability: New Perspectives on Physiological Mechanisms, Assessment of Self-regulatory Capacity, and Health risk. Glob. Adv. Health Med..

[57] Cohen L. (2002). Time-frequency distributions-a review. Proc. IEEE.

[58] Akselrod S., Gordon D., Ubel F.A., Shannon D.C., Berger A.C., Cohen R.J. (1981). Power spectrum analysis of heart rate fluctuation: A quantitative probe of beat-to-beat cardiovascular control. Science.

[59] Shaffer F., Ginsberg J.P. (2017). An Overview of Heart Rate Variability Metrics and Norms. Front. Public Health.

[60] Sztajzel J. (2004). Heart rate variability: A noninvasive electrocardiographic method to measure the autonomic nervous system. Swiss Med. Wkly..

[61] Galligan J.J., Parkman H. (2007). Recent advances in understanding the role of serotonin in gastrointestinal motility and functional bowel disorders. Neurogastroenterol. Motil..

[62] Grover M., Camilleri M. (2013). Effects on gastrointestinal functions and symptoms of serotonergic psychoactive agents used in functional gastrointestinal diseases. J. Gastroenterol..

[63] Crowell M.D. (2004). Role of serotonin in the pathophysiology of the irritable bowel syndrome. Br. J. Pharmacol..

[64] Atluri D.K., Chandar A.K., Fass R., Falck-Ytter Y. (2015). Systematic review with meta-analysis: Selective serotonin reuptake inhibitors for noncardiac chest pain. Aliment. Pharmacol. Ther..

[65] Thijssen A.Y., Mujagic Z., Jonkers D.M., Ludidi S., Keszthelyi D., Hesselink M.A., Clemens C.H., Conchillo J.M., Kruimel J.W., Masclee A.A. (2016). Alterations in serotonin metabolism in the irritable bowel syndrome. Aliment. Pharmacol. Ther..

[66] Wiśniewska-Jarosińska M., Harasiuk A., Klupińska G., Śmigielski J., Stec-Michalska K., Chojnacki C. (2010). Diagnostic value of measuring serum serotonin and urinary 5-hydroxyindoleacetic acid concentration in the diagnosis of functional dyspepsia. Gastroenterol. Rev..

[67] Bellono N.W., Bayrer J.R., Leitch D.B., Castro J., Zhang C., O’Donnell T.A., Brierley S.M., Ingraham H.A., Julius D. (2017). Enterochromaffin Cells Are Gut Chemosensors that Couple to Sensory Neural Pathways. Cell.

[68] Alcaino C., Knutson K.R., Treichel A.J., Yildiz G., Strege P.R., Linden D.R., Li J.H., Leiter A.B., Szurszewski J.H., Farrugia G. (2018). A population of gut epithelial enterochromaffin cells is mechanosensitive and requires Piezo2 to convert force into serotonin release. Proc. Natl. Acad. Sci. USA.

[69] Park D., Lee C., Woo D.H., Kim B., Yang Y.S., Song C.W. (2025). Transcriptome Features of Blood Samples in Patients Resembling Symptoms with Functional Dyspepsia. Korea Inst. Toxicol..

[70] Wang H., Kim S.J., Lei Y., Wang S., Wang H., Huang H., Zhang H., Tsung A. (2024). Neutrophil extracellular traps in homeostasis and disease. Signal Transduct. Target. Ther..

