# Supplementary material for: A Pilot Analysis of Bioparameters in Patients with Dyspepsia Accompanied by Abdominal Hardness: An Exploration of Damjeok Syndrome Rooted in Traditional Medicine
Source: Healthcare (Basel). 2025 Sep 15;13(18):2307. doi: 10.3390/healthcare13182307 (PMC12470122; doi:10.3390/healthcare13182307)
Supplement: Supplementary file 1 [file healthcare-13-02307-s001.zip › healthcare-3802598-supplementary.pdf]

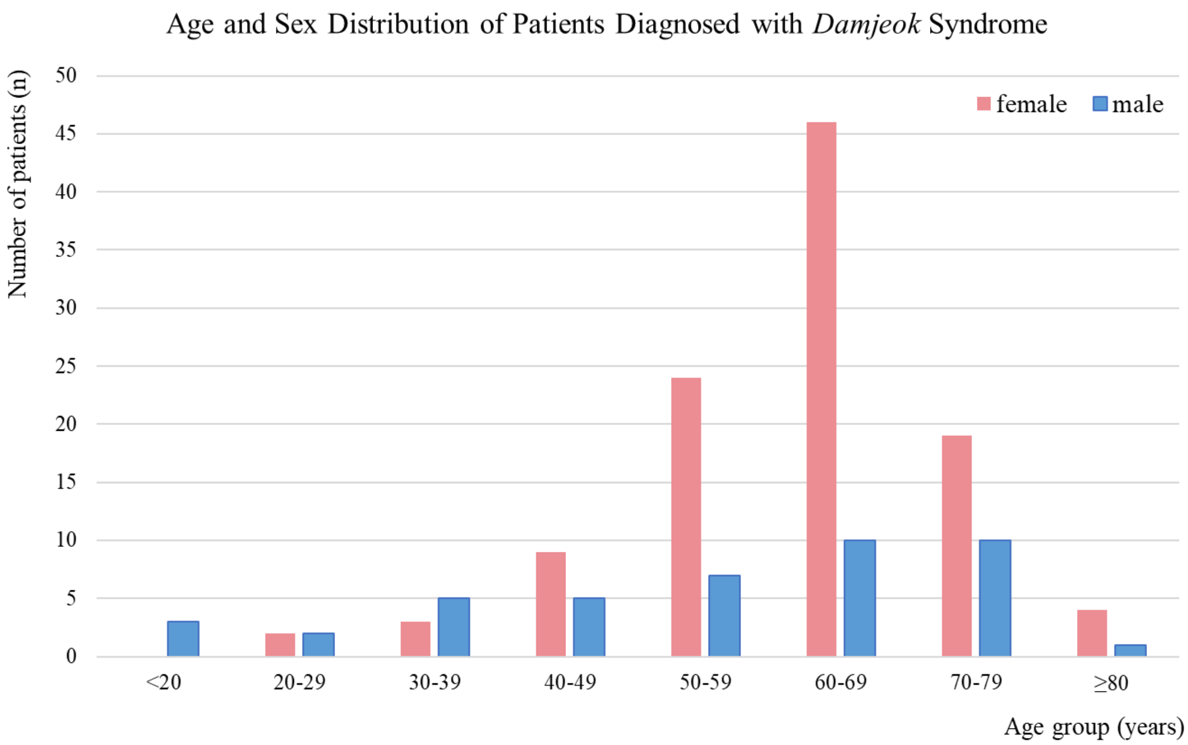

Supplementary Figure S1. Age and sex distribution of patients with *Damjeok* syndrome

Age and sex distribution of 150 patients diagnosed with *Damjeok* syndrome. Female patients accounted for 71.3% (n = 107), with the largest proportion in their 60s, followed by those in their 50s. Data were obtained from retrospective chart review at Weedahm Korean Medicine Hospital. This study was approved by the Institutional Review Board of Weedahm Korean Medicine Hospital (IRB No. WD00003-21-CR-001).
